# Chronic Kidney Disease Increases Mortality and Reduces the Chance of a Favorable Outcome in Stroke Patients Treated with Mechanical Thrombectomy—Single-Center Study

**DOI:** 10.3390/jcm13123469

**Published:** 2024-06-14

**Authors:** Michał Borończyk, Mikołaj Kuźniak, Agnieszka Borończyk, Kamil Barański, Anna Hawrot-Kawecka, Anetta Lasek-Bal

**Affiliations:** 1 Students’ Scientific Association, Department of Neurology, Faculty of Health Sciences in Katowice, Medical University of Silesia, 40-055 Katowice, Poland; mboronczyk@onet.pl (M.B.);; 2 Department of Epidemiology, Faculty of Medical Sciences in Katowice, Medical University of Silesia, 40-055 Katowice, Poland; 3 Department of Internal and Metabolic Diseases, Faculty of Health Sciences in Katowice, Medical University of Silesia, 40-752 Katowice, Poland; 4 Upper-Silesian Medical Centre, Medical University of Silesia, 40-752 Katowice, Poland; 5 Department of Neurology, Faculty of Health Sciences in Katowice, Medical University of Silesia, 40-752 Katowice, Poland

**Keywords:** renal impairment, chronic kidney disease, stroke, mechanical thrombectomy, outcomes

## Abstract

**Background/Objectives**: Chronic kidney disease (CKD) is identified as a risk factor for the occurrence of ischemic stroke. There is substantial evidence that CKD is linked to a worse prognosis and higher mortality rates in stroke patients. This study aimed to evaluate the characteristics and factors affecting favorable outcomes and mortality in patients treated using mechanical thrombectomy (MT) for ischemic stroke, with particular emphasis on patients suffering from CKD. **Methods**: The retrospective study included an analysis of data from 723 patients (139; 19.4% had CKD) with ischemic stroke treated with MT between March 2019 and July 2022. **Results**: Patients with CKD were significantly older (median age 76.5 vs. 65.65, *p* < 0.001) and more often female (59.7% vs. 42.6%, *p* < 0.001). CKD decreased the likelihood of achieving a favorable outcome (0–2 points in modified Rankin scale; OR: 0.56, CI95%: 0.38–0.81) and increased mortality (OR: 2.59, CI95%: 1.74–3.84) on the 90th day after stroke. In addition, CKD was associated with intracranial hemorrhage (ICH) in patients who underwent posterior circulation MT (13.85% vs. 50%, *p* = 0.022). In patients with CKD, inter alia, higher levels of C-reactive protein (OR: 0.94, CI95%: 0.92–0.99) reduced the chance of a favorable outcome. In addition, the occurrence of ICH in patients with CKD increased mortality on the 90th day after stroke (OR: 4.18, CI95%: 1.56–11.21), which was almost twice as high as in patients without CKD (OR: 2.29, CI95%: 1.54–3.40). **Conclusions**: Patients suffering from CKD had a lower probability of achieving a favorable outcome and had increased mortality following MT for ischemic stroke. It is crucial to understand the variations between patients with unimpaired and impaired renal function, as this could aid in predicting the outcomes of this method.

## 1. Introduction

Chronic kidney disease (CKD) and stroke have been listed among the top 25 leading causes of disability-adjusted life years (DALY)in recent years and, therefore, are considered as one of the leading health problems in the world [1]. Chronic kidney disease (CKD) and stroke share common cardiovascular risk factors, such as smoking, high blood pressure, dyslipidemia and diabetes mellitus (DM) [2]. Additionally, CKD is acknowledged as a risk factor for the occurrence of ischemic stroke [3,4,5,6]. One study revealed that an eGFR of less than 90 mL/min/1.73 m^2^ was linked to a 39% increased risk of all-cause stroke, with the risk escalating as renal function declines. In the study group with an eGFR of 60–90 mL/min/1.73 m^2^, the risk of stroke was increased by 10%, in participants with an eGFR of 30–60 mL/min/1.73 m^2^ by 43% and in participants with an eGFR of <30 mL/min/1.73 m^2^ by 70% [5]. Surprisingly, more than half of CKD patients are unaware of their condition on admission to the stroke unit [7].

There is ample evidence that CKD correlates with a poorer prognosis and increased mortality in patients hospitalized for stroke [3,5,8,9,10,11]. In addition, the incidence of intracranial hemorrhage (ICH) significantly increases in CKD patients after a thrombolytic treatment, with the risk of symptomatic ICH being 1.4–1.6 times higher than in patients without CKD [9,10]. In one study, Wang et al. proposed a dynamic nomogram as a prognostic tool for predicting adverse outcomes three months after stroke in patients with CKD, with an AUC (area under the curve) of 0.875 [12]. This nomogram, composed of eight independent factors, showed that mechanical thrombectomy (MT) reduced the likelihood of poor outcomes in this patient group (OR = 0.373, 95% CI: 0.145–0.964, *p* = 0.042).

MT is recognized as the gold standard method for the treatment of patients in the acute phase of large-vessel occlusion stroke [13]. Research demonstrates that CKD reduces the likelihood of a favorable prognosis, increases mortality and increases the risk of serious complications in stroke patients undergoing MT [14,15,16,17,18,19,20,21,22,23]. Additionally, it amplifies the risk of recurrent stroke [18] and may lead to post-contrast acute kidney injury, which occurs in 2.5–8.8% of patients after MT and is associated with a worse prognosis and an increased risk of death [11,24,25,26].

The ongoing debate revolves around the question of whether patients with CKD undergoing MT are more likely to develop ICH than patients with unimpaired renal function. Some studies suggest that CKD increases the likelihood of ICH [27,28], while other indicate that such a relationship does not exist [14,17,18]. Moreover, reports suggest that CKD increases the probability of bleeding in patients with posterior circulation MT (PMT) [29,30], while a study focusing exclusively on patients with anterior circulation MT (AMT) found no such observation [23].

The primary aim of this study was to assess the impact of specific clinical factors, particularly CKD, on the likelihood of favorable outcomes and mortality in patients undergoing MT for acute ischemic stroke. Additionally, the study sought to examine the factors influencing prognosis solely in a group of patients with CKD. Furthermore, the study aimed to compare differences between patients undergoing AMT and PMT, while considering the division of patients with normal and impaired renal function.

## 2. Materials and Methods

### 2.1. Patient Population

In this retrospective study, we conducted an analysis of data pertaining to patients who experienced ischemic stroke between 1 March 2019 and 31 July 2022 and received MT (stent retriever or contact aspiration as first-line technique) as the primary treatment. The subjects were sourced from the stroke subdivision of the Department of Neurology, Upper-Silesian Medical Centre of the Medical University of Silesia in Katowice, Poland. The data were retrieved from the hospital’s database. Patients with incomplete clinical data or those lost to follow-up were excluded from the study.

Patients were divided into two primary groups: those who underwent MT in the posterior circulation (PMT group), which included the vertebral artery (VA), basilar artery (BA) or posterior cerebral artery (PCA), and those who underwent MT in the anterior cerebral arteries (AMT group), which included the intracranial segments of the internal carotid artery (ICA), middle cerebral artery (MCA) or anterior cerebral artery (ACA).

Ethical approval was obtained from the local ethics committee, the “Bioethics Committee of the Medical University of Silesia in Katowice”, in accordance with the 1964 Declaration of Helsinki and its subsequent amendments. Due to the retrospective nature of the study, the need for informed consent was waived by the ethics committee.

### 2.2. Clinical Characteristics of the Cohort

We recorded the following patient characteristics: baseline demographic data such as age and sex, clinical data such as stroke severity (as measured using the National Institutes of Health stroke scale (NIHSS)) at admission and on the second day after stroke and risk factors such as atrial fibrillation (AF), arterial hypertension (AH), diabetes mellitus (DM), nicotinism, dyslipidemia and carotid artery stenosis (CAS). Patients were counted as having AH if they had a history of hypertension and were taking antihypertensive medication or if AH was identified for the first time during a hospitalization. Other comorbidities were identified based on the patients’ medical histories or diagnosed de novo during the hospital stay. Moreover, we obtained radiologic data on the localization of arterial occlusion, time from onset of symptoms to MT, efficacy of MT as measured using the modified thrombolysis in cerebral infarction (mTICI) scale (scores of 2b-3 were defined as successful and 0-2a as poor or absent reperfusion), the administration of IVT (alteplase at a dose of 0.9 mg/kg intravenously), laboratory test results, including creatinine, estimated glomerular filtration rate (eGFR), C-reactive protein (CRP), white blood cell (WBC) concentration, thrombocytosis (platelets ≥ 400.000/µL), thrombocytopenia (platelets ≤ 150.000/µL) and whether decompressive hemicraniectomy (DH) was performed.

The main criteria of the DH were as follows: Glasgow coma score > 5, NIHSS score ≥ 16, evidence of ischemic changes affecting more than 2/3 of the MCA territory on head computed tomography (CT) and the feasibility of intervention within 48 h of MT. Intracranial hemorrhage (ICH), encompassing the hemorrhagic transformation of ischemic stroke, was categorized in accordance with the European Cooperative Acute Stroke Study scale (ECASS II). Only type two parenchymal hematomas (PH2s) were included in the statistical analyses (>30% of the infarct zone, significant mass effect due to the hematoma).

Serum creatinine was measured on admission and monitored throughout hospitalization. If the creatinine level was normal on admission and the patient’s condition was stable, 2–3 measurements during hospitalization were usually sufficient. If the baseline eGFR was under 60 or the patient’s condition deteriorated, measurements were taken more frequently, on average every 2–3 days. The eGFR was assessed according to the CKD-EPI (Chronic Kidney Disease Epidemiology Collaboration) pattern. The presence of CKD was defined as an eGFR < 60 mL/min/1.73 m^2^ on admission, while patients with eGFR > 60 mL/min/1.73 m^2^ were defined as a non-renal impairment (nRI) group. In individuals with an eGFR < 60 mL/min/1.73 m^2^ (eGFR categories G3a-G5), we assessed medical histories and previous test results to determine the duration of renal disease, although we could not always obtain all the necessary information. Patients whose creatinine levels significantly improved or normalized during their hospital stay were excluded from the CKD group, as we assumed these cases were due to acute kidney injury. Serum creatinine levels were measured before contrast administration.

The functional outcomes of patients were assessed using the modified Rankin scale (mRS) at the following time points: before leaving the department and 30 and 90 days after stroke. A maximum score of two points on the mRS scale was considered a positive outcome. All previously mentioned components were compared between the PMT and AMT groups.

### 2.3. Statistical Analysis

Continuous variables were expressed as mean, median and range. Categorical variables were represented using numbers and percentages. The normality of distribution was assessed using the Shapiro–Wilk test. The significance of differences between groups for continuous variables was determined using either the Student t-test or the Mann–Whitney U test. Univariate analyses for categorical variables were performed using either the Chi-square test or Fisher exact test. Statistical significance was defined as a *p*-value < 0.05. A simple logistic regression analysis was used to determine the influence of individual phenodata on the dependent variables defined as mRS 0–2 vs. 3–6 (favorable outcome), mRS = 6 (mortality) and mTICI 2b-3 (successful reperfusion) at two time points, at discharge and on the 90th day after stroke, for the entire patient cohort and in the CKD group exclusively. The results were expressed as odds ratios (ORs) with a 95% confidence interval (CI95%). A statistical analysis was performed using Statistica 13.3 TIBCO Software Inc. 2024.

## 3. Results

### 3.1. Demographic and Clinical Characteristics of Patient Groups

**Figure 1:**
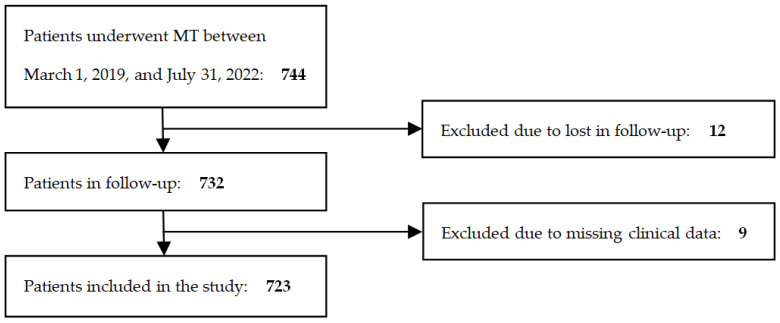
Protocol for qualifying patients for the study.

During the specified study period, 2642 patients were admitted to the Neurology Department at the Upper-Silesian Medical Centre of the Medical University of Silesia in Katowice for treatment of ischemic stroke. Among these patients, 744 underwent MT for the management of ischemic stroke. However, 12 patients were lost to follow-up and clinical data were found to be incomplete for an additional 9 individuals. Figure 1 depicts the protocol for patient qualification in the study.

The study included 723 stroke patients who underwent MT, with 648 (89.6%) in the AMT group and 75 (10.4%) in the PMT group. In the entire study population, 584 (80.8%) patients had unimpaired renal function (nRI group) and 139 (19.4%) patients had eGFR < 60 mL/min/1.73 m^2^ (CKD group). Within the AMT group, 519 (80.1%) patients had eGFR > 60 mL/min/1.73 m^2^ (nRI group) and 129 (19.9%) had eGFR < 60 mL/min/1.73 m^2^ (CKD group), while in the PMT group, 65 (86.7%) patients had eGFR > 60 mL/min/1.73 m^2^ (nRI group) and 10 (13.3%) had eGFR < 60 mL/min/1.73 m^2^ (CKD group). There were no significant differences in CKD occurrence between AMT and PMT patients.

The average age of the patients was 66.9, range: (20–92) years, with 44.9% of them being female. The patients in the AMT group differed significantly in terms of age (the median in the nRI group was 68 vs. 77 in the CKD group; *p* = 0.001), sex (42.6% women in the nRI group vs. 59.7% in the CKD group; *p* = 0.001) and comorbidities such as AF (34.9% in the nRI group vs. 51.9% in the CKD group; *p* = 0.001), AH (33.7% in the nRI group vs. 43.4% in the CKD group; *p* = 0.040), DM (22.7% in the nRI group vs. 34.9% in the CKD group; *p* = 0.004), CAS (67.8% in the nRI group vs. 85.3% in the CKD group; *p* = 0.001), nicotinism (27.4% in the nRI group vs. 9.3% in the CKD group; *p* = 0.001) and dyslipidemia (33.1% in the nRI group vs. 44.2% in the CKD group; *p* = 0.019). Moreover, in the AMT group, patients with CKD achieved worse outcomes at discharge (mean mRS 3.7 vs. 4.19, *p* = 0.036), on the 30th day after stroke (mean mRS 3.02 vs. 3.68; *p* = 0.001) and on the 90th day after stroke (mean mRS 2.67 vs. 3.57, *p* < 0.001). In addition, patients with CKD in the AMT group were less likely to achieve favorable outcomes on the 90th day after stroke (52.8% vs. 37.2%; *p* = 0.001), while a similar association was not observed either at discharge or on the 30th day time point. The same results were not observed in the PMT group, probably due to the small sample size.

**Figure 2:**
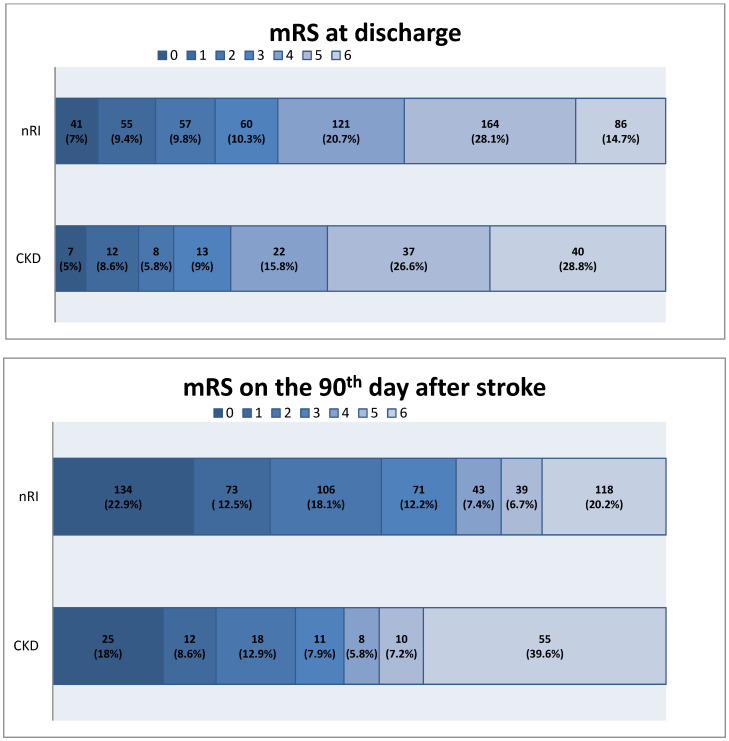
Comparison of mRS scores in patients with eGFR > 60 mL/min/1.73 m^2^ (nRI) and eGFR < 60 mL/min/1.73 m^2^ (CKD) at discharge and on the 90th day after stroke. eGFR: estimated glomerular filtration rate; mRS: modified Rankin scale; nRI: non-renal impairment; CKD: chronic kidney disease.

Patients within the PMT group differed significantly in terms of age (median in the nRI group = 63 vs. in the CKD group = 77; *p* = 0.001), AF (18.5% in the nRI group vs. 80% in the CKD group; *p* = 0.001) and CAS (23.1% in the nRI group vs. 60% in the CKD group; *p* = 0.041). Furthermore, patients with CKD in the PMT group had a significantly higher incidence of ICH (13.85% in the nRI group vs. 50% in the CKD group; *p* = 0.022) and had significantly higher rates of CRP levels (mean in the nRI group = 16.54 vs. in the CKD group = 29.91; *p* = 0.045) than those without renal impairment. Such relationships were not found in the AMT group. Comparative analyses between CKD patients in the PMT and the AMT groups revealed statistical differences only concerning CRP concentration (mean in the AMT group = 20.46 vs. in the PMT group = 29.91; *p* = 0.009). The incidence of ICH was not significantly higher in the PMT group than in the AMT group; however, the frequency was higher in the PMT patients (21.7% vs. 50%; *p* = 0.100). A complete comparative analysis is shown in Table 1. Figure 2 illustrates the distribution of mRS scores at discharge and on the 90th day after stroke between the normal and reduced eGFR groups.

### 3.2. Arterial Hypertension and Medications Used

We specified the type of antihypertensive treatment used by the patients. Of the 250 patients who suffered from AH, 211 (84.4%) used an antihypertensive treatment before the stroke, including 186 (74.4%) using polypharmacy, and in 39 (15.6%), AH was diagnosed during hospitalization and appropriate therapy was initiated. The most commonly used antihypertensive drugs were diuretics (in 153 patients; 61.2%) and angiotensin-converting enzyme inhibitors (in 112 patients; 44.8%). Other drugs used were calcium channel blockers in 81 patients (32.4%), angiotensin receptor antagonists in 31 patients (12.4%) and others in 29 patients (11.6%).

### 3.3. Predictors of Functional Independence

For the whole study cohort, we used the univariate logistic regression analysis to determine which factors influenced a favorable prognosis (mRS 0–2) at discharge and on the 90th day after stroke. Several factors reduced the likelihood of achieving favorable outcomes: older age, both at discharge (OR: 0.97, CI95%: 0.95–0.98) and on the 90th day after stroke (OR: 0.97, CI95%: 0.96–0.98); time of hospitalization, both at discharge (OR: 0.97, CI95%: 0.96–0.98) and on the 90th day after stroke (OR: 0.97, CI95%: 0.95–0.98); admission NIHSS score, both at discharge (OR: 0.85, CI95%: 0.82–0.89) and on the 90th day after stroke (OR: 0.88, CI95%: 0.85–0.91); AH only at discharge (OR: 0.66, CI95%: 0.46–0.95), DM only at discharge (OR: 0.50, CI95%: 0.32–0.78); white blood cell concentration, both at discharge (OR: 0.84, CI95%: 0.80–0.89) and on the 90th day after stroke (OR: 0.91, CI95%: 0.87–0.95); CRP serum concentration, both at discharge (OR: 0.98, CI95%: 0.97–0.99) and on the 90th day after stroke (OR: 0.98, CI95%: 0.98–0.99); time of MT, both at discharge (OR: 0.98, CI95%: 0.98–0.99) and on the 90th day after stroke (OR: 0.99, CI95%: 0.98–0.99); time from symptoms to MT only at discharge (OR: 0.99, CI95%: 0.98–0.99); and ICH, both at discharge (OR: 0.41, CI95%: 0.24–0.69) and on the 90th day after stroke (0.56, CI95%: 0.37–0.86).

The only factors increasing the likelihood of favorable outcomes were thrombosis in a posterior cerebral artery at discharge (OR: 2.41, CI95%: 1.04–5.60) and successful recanalization, both at discharge (OR: 4.59, CI95%: 2.58–8.18) and on the 90th day after stroke (OR: 2.47, CI95%: 1.70–3.60). Neither CKD nor the creatinine level showed to be significant for the prognosis at discharge; however, patients with CKD were less likely to achieve favorable outcomes on the 90th day after stroke (OR: 0.56, CI95%: 0.38–0.81). Table 2 depicts this analysis.

Additionally, the analysis conducted only in a group of patients with eGFR < 60 mL/min/1.73 m^2^ showed that older age reduced the chances of a favorable outcome at discharge more than in the entire population (OR: 0.94, CI95%: 0.90–0.99). The analysis also found that a longer hospital stay strongly reduced the probability of favorable outcomes on the 90th day after stroke (OR: 0.95, CI95%: 0.92–0.99) compared to in the overall study cohort. Furthermore, in CKD patients, higher CRP levels were associated with a worse prognosis (OR: 0.94, CI95%: 0.92–0.99), and this relationship was also stronger than in the entire cohort.

### 3.4. Predictors of Mortality

We applied the same model to specify which factors impacted mortality at discharge and on the 90th day after stroke. Factors that increased mortality included the following: older age, both at discharge (OR: 1.03, CI95%: 1.01–1.04) and on the 90th day after stroke (OR: 1.03, CI95%: 1.01–1.05); admission NIHSS score, both at discharge (OR: 1.09, CI95%: 1.05–1.13) and on the 90th day after stroke (OR: 1.08, CI95%: 1.04–1.12); DM, both at discharge (OR: 1.75, CI95%: 1.16–2.64) and on the 90th day after stroke (OR: 1.63, CI95%: 1.09–2.45); thrombosis in a BA, both at discharge (OR: 2.30, CI95%: 1.18–4.46) and on the 90th day after stroke (OR: 2.25, CI95%: 1.21–4.19); WBC concentration, both at discharge (OR: 1.09, CI 95%: 1.04–1.14) and on the 90th day after stroke (OR: 1.07, CI95%: 1.02–1.12); CRP concentration only at discharge (OR: 1.006, CI95%: 1.001–1.012); time of MT, both at discharge (OR: 1.004, CI95%: 1.000–1.009) and on the 90th day after stroke (OR: 1.006, CI95%: 1.002–1.011); time from symptoms to MT only at discharge (OR: 1.002, CI95%: 1.000–1.004); and the occurrence of ICH, both at discharge (OR: 2.22 CI95%: 1.44–3.42) and on the 90th day after stroke (OR: 2.03, CI95%: 1.03–3.18).

There were also factors that reduced the chances of mortality, including the following: time of hospitalization at discharge (OR: 0.98, CI95%: 0.96–1.00) and mTICI 2b-3 at discharge (OR: 0.60, CI95%: 0.39–0.92) and on the 90th day after stroke (OR: 0.48, CI95%: 0.32–0.72). Furthermore, both CKD and creatinine levels increased the chance of death, both at discharge (for CKD (OR: 2.34; CI95%: 1.52–3.61) and for creatinine levels (OR: 2.16; CI95%: 1.34–3.49)) and on the 90th day after stroke (for CKD (OR: 2.59; CI95%: 1.74–3.84) and for creatinine levels (OR: 2.38; CI95%: 1.38–3.81)). The full analysis is shown in Table 3.

In the group of patients with CKD exclusively, the factor influencing both mortality at discharge (OR: 4.13; CI95%: 1.78–9.59) and on the 90th day after stroke (OR: 4.18; CI95%: 1.56–11.21) was the occurrence of ICH, and the risk of death was almost twice as high as in the entire cohort.

### 3.5. Predictors of Endovascular Revascularization in Patients with Chronic Kidney Disease

We also used a univariate regression analysis on the grade of revascularization after MT in patients with eGFR < 60 mL/min/1.73 m^2^ exclusively (Table 4). Older age (OR: 0.98; CI95%: 0.96–0.99), AF (OR: 0.60; CI95%: 0.42–0.86), CAS (OR: 0.49; CI95%: 0.34–0.69) and a longer time of MT (OR: 0.985; CI95%: 0.981–0.989) were associated with an unsuccessful recanalization result in CKD patients.

## 4. Discussion

Our study showed that a reduced eGFR was a poor prognostic factor (OR: 0.56 for a favorable outcome on the 90th day after stroke) and, most importantly, increased mortality in stroke patients undergoing MT (OR 2.34 at discharge and 2.59 on the 90th day after stroke). According to previous observations, a reduced eGFR on admission in stroke patients serves as an unfavorable prognostic factor that occurs in endovascularly treated patients [14,15,18,21,22,23,27], as well as in all stroke patients [8,9]. In a study conducted by El Husseini et al. involving an analysis of 232,236 patients, eGFR levels below 60 mL/min/1.73 m^2^ were found to be independently associated with heightened odds of in-hospital mortality or discharge to hospice, with the highest risk observed among those with eGFR < 15 mL/min/1.73 m^2^ without dialysis. Furthermore, the study indicated that stroke patients with eGFR < 60 mL/min/1.73 m^2^ tended to be older than those with eGFR > 60 mL/min/1.73 m^2^ [31].

In line with this observation, in our study, patients with eGFR < 60 mL/min/1.73 m^2^ were older in both the AMT and PMT groups, which in itself is a risk factor for a poorer prognosis [15,32,33,34]. The independent association of CKD with a poorer prognosis may be explained through uremic toxins. These toxins can contribute to a disruption in the blood–brain barrier, a disturbed coagulation and thrombosis balance and endothelial dysfunction [3,35]. In addition, CKD is associated with a systemic inflammatory state [36,37], which may manifest as local neuroinflammation [3].

In our study, CRP levels were significantly higher in the group of PMT patients with CKD compared to those with a normal eGFR and showed a difference between the AMT and PMT groups, a relationship that we believe was novel. Conversely, PMT was previously associated with a more intense inflammatory response [38,39,40]. Furthermore, in our previous study, we showed that in patients with PMT, both higher CRP levels (OR: 0.995, CI95%: 0.992–0.999) and leukocytosis (OR: 0.974, CI95%: 0.957–0.998) were independent and unfavorable prognostic factors [41].

The occurrence of ICH was found to be a significant independent prognostic factor in the group of patients with a reduced eGFR, increasing both in-hospital mortality (OR: 4.13) and mortality on the 90th day after stroke (OR: 4.18). This impact was almost twice as high as in the entire study group (OR: 2.22 and 2.03, respectively). ICH is a recognized risk factor for an unfavorable prognosis in patients after MT, increasing mortality as well [33,42,43]. This is reinforced by the fact that patients with CKD have a significantly increased risk of bleeding due to impaired platelet function and anemia [44,45], which may explain why the risk of bleeding complications is even greater in this patient group.

In a study by Tzu-Hao Chao on patients with eGFR < 60 mL/min/m^2^ treated with IVT, ICH (asymptomatic, symptomatic or petechial) occurred significantly more frequently in the group with a reduced eGFR (23 vs. 12.5%, *p*< 0.05) [46]. In another study, Yoav Arnson et al. showed that with an increasing severity of CKD, both bleeding susceptibility (the average HAS-BLEED score of patients in stage 1 was 1.0 ± 0.9 and in stage 4–5 2.3 ± 0.7) and the incidence of a history of ICH increased (0.8% in stage 1 versus 2% in stages 4–5) [47]. Furthermore, the same study showed that CKD stages 4–5 were associated with a hazard ratio (HR) = 1.59 (CI95%: 1.17–2.15) for ICH in patients with non-valvular AF compared to patients with normal renal function [47]. In our patient group, the incidence of ICH in patients with a reduced eGFR was significantly higher only in the PMT group. This observation was consistent with the literature data reporting the incidence of ICH in patients with a reduced eGFR undergoing PMT. In a study by Lulu Xiao, eGFR < 60 mL/min/1.73 m^2^ was independently associated with symptomatic ICH in patients undergoing PMT (OR: 3.30, CI95%: 1.55–7.18). Interestingly, in the subgroup of patients receiving direct MT, the OR was as high as 5.53 (CI95%: 2.34–13.07), but no statistical significance was found in the case of bridging therapy (IVT + MT) [29].

Another study by Mona Laible showed that both eGFR < 60 mL/min/1.73 m^2^ (OR: 3.73, CI95%: 1.20–11.60) and the presence of DM (OR: 4.56, CI95%: 1.42–14.62) increased the likelihood of ICH in patients with PMT [30]. In the same study, researchers determined that the occurrence of ICH in patients with PMT and eGFR < 60 mL/min/1.73 m^2^ increased mortality by more than fourfold (OR: 4.05, CI95%: 1.21–13.58) [30]. It is important to note that some studies showed a higher incidence of ICH in patients with PMT, regardless of their creatinine concentrations [48,49]. In studies that did not differentiate between AMT and PMT or that only considered AMT patients, there was generally no difference in ICH between patients with a reduced and normal eGFR [14,15,17,18,23,27]. Nevertheless, it was observed that patients on dialysis with CKD had a higher incidence of ICH (15.0%) than those with CKD without dialysis (3.6%) [27]. These observations suggested that patients with impaired renal function and PMT are potentially at the highest risk of developing ICH complications.

Moreover, in our analysis, we demonstrated that there is a relationship between factors such as age, AF, AO, and MT time and successful recanalization in patients with CKD. To our knowledge, we were the first to describe these associations.

Patients with CKD were significantly more likely to suffer from all of the comorbidities we examined, including AF, AH, DM, dyslipidemia and CAS. AF is a condition associated with CKD in stroke patients in most studies [14,17,18,22,27,28,29,30,47,50,51]. Patients with a concomitant AF and lower eGFR have an increased risk of stroke [52,53], as well as poorer outcomes, higher mortality and stroke recurrence [12,50,54] after a stroke treatment. Wang et al. showed in their study that a reduced eGFR more than doubled the risk of death in patients with ischemic stroke and AF 3, 6 and 12 months after stroke (OR: 2.203; CI95%: 1.235–3.929 on the 12th month) [54]. The mechanisms explaining why CKD is associated with AF are not fully understood; an increased likelihood of developing AH, fluid overload and the pathologic activation of the renin–angiotensin–aldosterone system leading to myocardial fibrosis are thought to play an important role [55].

In patients with CKD treated with MT due to stroke, DM occurred more frequently, as confirmed by numerous other authors [14,17,18,22,27,29,30]. In one study, the risk of stroke in patients with DM increased with the progression of CKD from stage G1 to G3, reaching the highest value for CKD G3 in the under 62 years old group (HR: 3.12, CI95%: 1.02–9.56) [51]. It has been postulated that the increased stroke burden in people with DM and CKD is largely due to the more common metabolic syndrome in these patients [56] and the numerous microvascular complications associated with DM [57,58].

Another parameter that was significantly higher in the CKD group was dyslipidemia and the associated atherosclerosis leading to CAS. This finding was consistent with previous research studies [14,17,22,30,47,51]. In one study, 92.7% of patients with reduced kidney function treated with MT had hyperlipidemia [22]. Another study in a Chinese population found that patients with polyvascular arteriosclerosis and stroke had a significantly higher incidence of CKD [59]. However, in another study, a history of dyslipidemia was associated with in-hospital mortality only in patients with an eGFR of 30–44 mL/min/1.73 m^2^ [31]. Treatment with statins during hospitalization for stroke in patients with renal impairment reduced mortality and the likelihood of a poor prognosis [60,61,62]. A meta-analysis showed that a statin treatment in CKD patients reduced the risk of stroke by 30% (risk ratio = 0.70; CI95%: 0.57–0.85) [62]. Furthermore, the treatment with simvastatin plus ezetimibe resulted in a significant reduction in non-hemorrhagic strokes (2.8% vs. 3.8%), mainly due to a significant reduction in definite ischemic strokes. Nonetheless, there was a significant reduction in the risk of any type of stroke (3.7% vs. 4.5%) [63].

AH is another common comorbidity in CKD patients treated for stroke [14,17,18,22,27,29,30,47,51]. Patients with AH and CKD have been shown to be more prone to poor outcomes after MT [16]. In the study by Laible et al. in patients with posterior circulation stroke and a reduced eGFR, 90.9% of the patients had AH, and its occurrence was associated with an OR of 3.19 (CI95%: 1.21–8.36) for poor outcomes (3–6 mRS) [30].

These considerations lead to the conclusion that patients with CKD treated for stroke are a patient group with a significantly higher burden of comorbidities that further worsen neurological outcomes. Therefore, special attention should be paid to the treatment of both the underlying disease and comorbidities in this patient group.

### Limitations of the Study

We acknowledge the limitations inherent in our study that necessitate attention. Firstly, the admission and qualification for MT were overseen by different physicians, potentially leading to variances in the interpretation of clinical and radiological symptoms. Secondly, it is recognized that patients underwent MT were either directly admitted to our center or transferred from a primary center utilizing the drip-and-ship model. This circumstance may have introduced disparities in MT qualification and the duration between MT and IVT administration. Thirdly, our study predominantly relied on the clinical status of patients at discharge and on the 90th day post-stroke, whereas some investigations extended their focus to outcomes after one year. Fourthly, the utilization of the NIHSS scale to assess the clinical condition of patients undergoing AMT and PMT procedures may not have comprehensively captured the severity of the clinical condition in patients with posterior circulation stroke. Fifthly, we used the modified Rankin scale to assess functional status, which has its limitations, while other clinical functional assessment scales, such as the functional independence measure or Barthel’s scale, were also available. Sixthly, outcomes and mortality could have been influenced by other factors, such as the occurrence of pneumonia, Clostridium difficile infection and others. Seventhly other factors, such as social support of patients, could have influenced the assessment of outcomes after stroke using mRS. Finally, the retrospective nature of our study and the relatively small size of the subgroup of patients undergoing PMT may have potentially compromised the robustness of our results. Therefore, we suggest that future studies be conducted with a larger number of patients with posterior cerebrovascular stroke to further our understanding in this area.

## 5. Conclusions

Our study confirmed that patients with a reduced eGFR who underwent MT had a lower chance of achieving favorable outcomes and a higher mortality rate, which was consistent with findings from previous studies. Additionally, we showed that CKD is associated with a higher incidence of comorbidities, such as AH, DM, AF and dyslipidemia. Moreover, we identified several factors that reduced the odds of favorable outcomes and increased mortality in patients with CKD more than in the overall study population, including age, CRP levels and occurrence of ICH. Furthermore, we compared the patient populations undergoing AMT and PMT and found that ICH was significantly more common in the PMT group. However, it should be noted that the population of PMT patients in our study was small due to the rarity of posterior circulation strokes, which may have masked more significant differences between these patient populations. Therefore, conducting a larger number of comprehensive studies on this topic is crucial to adequately characterize the risk factors and dependencies in this patient population. This may help optimize therapy for this patient cohort in the future.

## Figures and Tables

**Table 1:** Baseline characteristics of patients with mechanical thrombectomy in anterior (AMT) and posterior (PMT) cerebral circulation, divided into patients with eGFR > 60 mL/min/1.73 m^2^ (nRI) and eGFR < 60 mL/min/1.73 m^2^ (CKD).

	AMT (n = 648)			PMT (n = 75)			
	eGFR > 60 (nRI) (n = 519)	eGFR < 60 (CKD) (n = 129)	*p*-Value AMT + nRI vs. AMT + CKD	eGFR > 60 (nRI) n = 65	eGFR < 60 (CKD) n = 10	*p*-Value PMT + nRI vs. PMT + CKD	*p*-Value AMT + CKD vs. PMT + CKD
Age (mean, median, range)	65.65, 68, [19, 89]	76.52, 77, [48, 92]	**<0.001** ^1^	61.54, 63, [23, 89]	75.6, 77, [55, 91]	**<0.001** ^1^	0.763 ^1^
Female (n, %)	221 (42.6%)	77 (59.7%)	**<0.001** ^2^	18 (27.7%)	5 (50%)	0.291 ^3^	0.790 ^3^
Hospitalization time (mean, median, range)	12.06, 9, [1, 70]	12.67, 9, [1, 60]	0.823 ^1^	10.38, 8, [0, 55]	11.6, 11, [2, 20]	0.178^1^	0.660 ^1^
Early ischemic changes in CT (n, %)	211 (40.7%)	64 (49.6%)	0.166 ^2^	19 (29.2%)	4 (40%)	0.750 ^3^	0.800 ^3^
NIHSS1 (mean, median, range)	13.25, 13 [1, 30]	13.92, 14, [3, 29]	0.279 ^1^ **0.033** ^2^	11.4, 10, [1, 43]	11.8, 11.5, [4, 22]	0.691 ^1^	0.273 ^1^
NIHSS2 (mean, median, range)	12.31, 12, [0, 30]	12.95, 13, [0, 29]	0.404 ^1^ 0.740 ^2^	11.52, 7, [0, 43]	12.7, 13, [0, 26]	0.503 ^1^	0.935 ^1^
mRS at discharge (mean, median, range)	3.7, 4, [0, 6]	4.19, 5, [0, 6]	**0.036** ^2^	3.58, 4, [0, 6]	3.9, 4.5, [0, 6]	0.059 ^2^	0.115 ^3^
mRS at discharge (0–2) (n, %)	132 (25.4%)	25 (19.4%)	0.594 ^2^	20 (30.8%)	2 (20%)	0.746 ^3^	0.714 ^3^
mRS at 1 month (mean, median, range)	3.02, 3, [0, 6]	3.68, 4, [0, 6]	**0.001** ^2^	3.12, 3, [0, 6]	3.00, 3, [0, 6]	0.674 ^2^	0.595 ^2^
mRS at 1 month (0–2) (n, %)	208 (40.1%)	41 (31.8%)	0.083 ^2^	27 (41.5%)	4 (40%)	0.800 ^3^	0.853 ^3^
mRS at 3 months (mean, median, range)	2.67, 2, [0, 6]	3.57, 4, [0, 6]	**<0.001** ^2^	2.93, 2, [0, 6]	3.10, 3, [0, 6]	0.560 ^2^	0.786 ^2^
mRS at 3 months (0–2) (n, %)	274 (52.8%)	48 (37.2%)	**0.001** ^2^	32 (49.2%)	4 (40%)	0.838 ^3^	0.870 ^3^
AF (n, %)	181 (34.9%)	67 (51.9%)	**<0.001** ^2^	12 (18.5%)	8 (80%)	**<0.001** ^3^	0.101 ^3^
AH (n, %)	175 (33.7%)	56 (43.4%)	**0.040** ^2^	15 (23.1%)	4 (40%)	0.450 ^3^	0.954 ^3^
DM (n, %)	118 (22.7%)	45 (34.9%)	**0.004** ^2^	40 (61.5%)	9 (90%)	0.160 ^3^	0.492 ^3^
CAS (n, %)	352 (67.8%)	110 (85.3%)	**<0.001** ^2^	15 (23.1%)	6 (60%)	**0.041** ^3^	0.986 ^3^
Smoking (n, %)	142 (27.4%)	12 (9.3%)	**<0.001** ^2^	19 (29.2%)	1 (10%)	0.221 ^3^	0.300 ^3^
Dyslipidemia (n, %)	172 (33.1%)	57 (44.2%)	**0.019** ^2^	25 (38.5%)	2 (20%)	0.436 ^3^	0.247 ^3^
IVT (n, %)	336 (64.7%)	78 (60.5%)	0.366 ^2^	49 (75.4%)	5 (50%)	0.198 ^3^	0.752 ^3^
mTICI 2b-3 (n, %)	409 (78.8%)	93 (72.1%)	0.102 ^2^	53 (81.5%)	5 (50%)	0.07 ^3^	0.264 ^3^
ICH (n, %)	97 (18.7%)	28 (21.7%)	0.437 ^2^	9 (13.85%)	5 (50%)	**0.022** ^3^	0.100 ^3^
Hemicraniectomy (n, %)	24 (4.6%)	4 (3.1%)	0.444 ^2^	3 (4.6%)	0 (0%)	0.862 ^3^	0.677 ^3^
Time from symptoms to MT (groin puncture) (mean, median, range)	271.54, 270, [5, 870]	261.44, 267, [60, 660]	0.198 ^1^	278.43, 267, [98, 780]	315, 320, [150, 556]	0.201 ^1^	0.084 ^1^
MT time (mean, median, range)	95.88, 90, [30, 270]	98.05, 90, [30, 225]	0.722 ^1^	101.03, 100, [45, 190]	92.2, 85, [57, 150]	0.436 ^1^	0.627 ^1^
CRP concentration (mean, median, range)	20.02, 8.5, [3, 254]	20.46, 9, [3, 254]	0.359 ^1^	16.54, 8, [4, 127]	29.91, 21.5, [5, 72]	**0.045** ^1^	**0.009** ^1^
WBC (mean, median, range)	11.05, 10, [4.63, 88]	10.79, 10, [3, 22]	0.943 ^1^	10.77, 10.75, [5, 17]	9.94, 10.41, [5.5, 15]	0.454 ^1^	0.613 ^1^
Thrombocytosis (n, %)	11 (2.1%)	4 (3.1%)	0.292 ^2^	2 (3.1%)	0 (0%)	0.623 ^3^	0.720 ^3^
Thrombocytopenia (n, %)	37 (7.2%)	14 (10.85%)	0.318 ^2^	3 (4.6%)	2 (20%)	0.256 ^3^	0.677 ^3^

AMT: anterior mechanical thrombectomy; PMT: posterior mechanical thrombectomy; eGFR: estimated glomerular filtration rate; CT: computed tomography; NIHSS1: National Institutes of Health stroke scale on admission; NIHSS2: National Institutes of Health stroke scale 24 h after intervention; mRS: modified Rankin scale; AF: atrial fibrillation; AH: arterial hypertension; DM: diabetes mellitus; CAS: carotid artery stenosis; IVT: intravenous thrombolysis; mTICI 2b-3: modified thrombolysis In cerebral infarction scale 2b-3; ICH: intracranial hemorrhage; MT: mechanical thrombectomy; CRP: C-reactive protein; WBC: white blood cells. ^1^ U Mann–Whitney test; ^2^ Pearson’s Chi^2^test; ^3^ Yates’ Chi^2^test. Bold indicates statistical significance.

**Table 2:** Logistic regression analysis of clinical parameters that impacted on receiving less than 3 points in mRS at discharge and on the 90th day after stroke in the entire study cohort.

	Functional Independence at Discharge (mRS 0–2)	Functional Independence on 90th Day (mRS 0–2)
Predictors	OR and CI95%	OR and CI95%
Age	**0.97 (0.95–0.98)**	**0.97 (0.96–0.98)**
Female	1.07 (0.76–1.51)	0.85 (0.62–1.17)
Time of the hospitalization	**0.97 (0.96–0.98)**	**0.97 (0.95–0.98)**
NIHSS1	**0.85 (0.82–0.89)**	**0.88 (0.85–0.91)**
AF	0.79 (0.55–1.13)	0.81 (0.59–1.12)
AH	**0.66 (0.46–0.95)**	0.88 (0.62–1.24)
DM	**0.50 (0.32–0.78)**	0.77 (0.52–1.07)
CAS	0.95 (0.67–1.36)	0.76 (0.55–1.05)
Dyslipidemia	0.94 (0.66–1.35)	1.33 (0.74–1.43)
ICA	0.71 (0.45–1.12)	0.79 (0.53–1.17)
ACA	1.19 (0.54–2.64)	1.03 (0.43–2.42)
MCA	1.19 (0.79–1.77)	1.17 (0.81–1.70)
VA	1.07 (0.41–2.76)	1.18 (0.47–2.99)
BA	0.86 (0.41–1.77)	0.80 (0.39–1.61)
PCA	**2.41(1.04–5.60)**	1.89 (0.71–5.05)
WBC	**0.84 (0.80–0.89)**	**0.91 (0.87–0.95)**
CRP concentration	**0.98 (0.97–0.99)**	**0.98 (0.98–0.99)**
MT time	**0.98 (0.98–0.99)**	**0.99 (0.98–0.99)**
Time from symptoms to MT	**0.99 (0.98–0.99)**	0.99 (0.99–1.00)
mTICI 2b-3	**4.59 (2.58–8.18)**	**2.47 (1.70–3.60)**
ICH	**0.41 (0.24–0.69)**	**0.56 (0.37–0.86)**
CKD	0.69 (0.43–1.08)	**0.56 (0.38–0.81)**
Creatinine level	0.91 (0.57–1.47)	0.69 (0.45–1.05)

mRS: modified Rankin scale; OR: odds ratio; CI: confidence interval; NIHSS1: National Institutes of Health stroke scale on admission; AF: atrial fibrillation; AH: arterial hypertension; DM: diabetes mellitus; CAS: carotid artery stenosis; ICA: internal carotid artery; ACA: anterior cerebral artery; MCA: medium cerebral artery; VA: vertebral artery; BA: basilar artery; PCA: posterior cerebral artery; CRP: C-reactive protein; WBC: white blood cell count; mTICI 2b-3: modified thrombolysis in cerebral infarction scale 2b-3; ICH: intracranial hemorrhage; CKD: chronic kidney disease. Bold indicates statistical significance.

**Table 3:** Logistic regression analysis of clinical parameters that impacted mortality at discharge and on the 90th day after stroke in the entire study cohort.

	Mortality at Discharge	Mortality at 90th Day
Predictors	OR and CI95%	OR and CI95%
Age	1.03 (1.01–1.04)	1.03 (1.01–1.05)
Female	**1.14 (0.78–1.68)**	**0.85 (0.62–1.17)**
Time of the hospitalization	0.98 (0.96–1.00)	1.01 (0.99–1.02)
NIHSS1	**1.09 (1.05–1.13)**	1.08 (1.04–1.12)
AF	**1.18 (0.79–1.74)**	**0.81 (0.59–1.12)**
AH	1.12 (0.73–1.72)	0.88 (0.62–1.24)
DM	**1.75 (1.16–2.64)**	**1.63 (1.09–2.45)**
CAS	1.16 (0.78–1.73)	0.76 (0.55–1.05)
Dyslipidemia	1.29 (0.87–1.92)	1.03 (0.74–1.43)
ICA	0.97 (0.59–1.59)	0.79 (0.53–1.17)
ACA	1.93 (0.87–4.29)	1.03 (0.43–2.42)
MCA	0.86 (0.56–1.33)	1.17 (0.81–1.70)
VA	1.00 (0.33–3.01)	1.89 (0.72–2.99)
BA	**2.30 (1.18–4.46)**	**2.25 (1.21–4.19)**
PCA	1.15 (0.34–3.86)	1.89 (0.71–5.05)
WBC	**1.09 (1.04–1.14)**	**1.07 (1.02–1.12)**
CRP concentration	**1.006 (1.001–1.012)**	1.003 (0.997–1.009)
MT time	**1.004 (1.000–1.009)**	**1.006 (1.002–1.011)**
Time from symptoms to MT	**1.002 (1.000–1.004)**	1.002 (1.000–1.003)
mTICI 2b-3	**0.60 (0.39–0.92)**	**0.48 (0.32–0.72)**
ICH	**2.22 (1.44–3.42)**	**2.03 (1.03–3.18)**
CKD	**2.34 (1.52–3.61)**	**2.59 (1.74–3.84)**
Creatinine level	**2.16 (1.34–3.49)**	**2.38 (1.38–3.81)**

OR: odds ratio; CI: confidence interval; NIHSS1: National Institutes of Health stroke scale on admission; AF: atrial fibrillation; AH: arterial hypertension; DM: diabetes mellitus; CAS: carotid artery stenosis; ICA: internal carotid artery; ACA: anterior cerebral artery; MCA: medium cerebral artery; VA: vertebral artery; BA: basilar artery; PCA: posterior cerebral artery; CRP: C-reactive protein; WBC: white blood cell count; MT: mechanical thrombectomy; mTICI 2b-3: modified thrombolysis in cerebral infarction scale 2b-3; ICH: intracranial hemorrhage; CKD: chronic kidney disease. Bold indicates statistical significance.

**Table 4:** The effect of clinical phenodata on reperfusion effectiveness as measured using TICI 2b-3 in CKD patients.

	mTICI 2b-3
Predictors	OR and CI95%
Age	0.98 (0.96–0.99)
AF	0.60 (0.42–0.86)
CAS	0.49 (0.34–0.69)
Time of MT	0.985 (0.981–0.989)

mTICI 2b-3: modified thrombolysis in cerebral infarction scale 2b-3; OR: odds ratio; CI: confidence interval; AF: atrial fibrillation; CAS: carotid artery stenosis; MT: mechanical thrombectomy.

## Data Availability

The datasets generated and analyzed during the study are not publicly available because they contain personal data of the patients, but they can be requested from the corresponding author upon reasoned request.
